# Geospatial and hot spot analysis of paediatric tuberculosis infection in Bohol, Philippines

**DOI:** 10.1017/S0950268820000795

**Published:** 2020-05-06

**Authors:** L. M. Leining, S. R. Gatchalian, S. M. Gunter, N. T. Castillo-Carandang, A. M. Mandalakas, A. T. Cruz, J. B. McCormick, K. O. Murray

**Affiliations:** 1 Department of Paediatrics, Baylor College of Medicine and Texas Children's Hospital, Houston, TX, USA; 2 Division of Epidemiology, Human Genetics and Environmental Sciences, University of Texas Health Science Centre at Houston, School of Public Health, Houston, TX, USA; 3 Department of Paediatrics, College of Medicine, Philippine General Hospital, University of the Philippines Manila, Philippines; 4 Department of Clinical Epidemiology, College of Medicine, and Institute of Clinical Epidemiology, National Institutes of Health, University of the Philippines Manila, Philippines; 5 Division of Epidemiology, Human Genetics and Environmental Sciences, Brownsville Regional Campus, University of Texas Health Science Centre, School of Public Health, Brownsville, TX, USA

**Keywords:** geospatial analysis, hot spot analysis, paediatric, Philippines, tuberculosis

## Abstract

Tuberculosis (TB) in children is a critical public health issue. In Bohol, Philippines, we found a high tuberculin skin test (TST)-positive prevalence (weighted prevalence = 6.4%) among 5476 children (<15 years) from 184 villages, with geographically isolated communities having prevalence as high as 29%. Therefore, we conducted a geospatial and hot spot analysis to examine the association between villages with high TST-positive prevalence (⩾6.5%) and access to medical care (distance (in kilometres and minutes of travel time) to the municipal Rural Health Units (RHU)), access to healthcare resources (distance to Provincial Health Office (PHO)) and socioeconomic determinants of health. Hot spot analysis revealed significant clusters of TST-positive prevalence in villages farthest from the PHO. Based on univariate analysis, the following variables associated with high prevalence were included in the multivariate model: minutes of travel time to the PHO, distance to the PHO, island villages and total deprivation based on socioeconomic indicators. In the final model, only distance to PHO in minutes was significant (*P* = 0.005). When evaluated further, greater than 1-hour drive significantly increased risk for TST-positivity (*P* = 0.003). Distance to healthcare resources likely increases the risk of TB transmission within the community. Expanding TB control efforts to geographically isolated areas is critical.

## Introduction

Tuberculosis (TB) is a communicable infectious disease spread through aerosolised droplets of *Mycobacterium tuberculosis* [1]. In 2017, TB was one of the top ten causes of adult mortality in the world; among children, it was responsible for an estimated one million cases of incident TB and 253 000 reported deaths [2]. The Philippines has the second highest incidence in the world, accounting for 6% of all global cases [3, 4]. Reported Philippine national TB estimates of paediatric cases in 2016 were 613 cases per 100 000 (95% CI = 403–822) among individuals 15–19 years old [5]. Prior to our study, there were no known reports of estimates of TB prevalence or incidence in children under age 15 in the Philippines. Addressing paediatric TB is important because children are exposed to adults with active TB and are vulnerable to develop severe disease [6, 7]. Additionally, they can serve as indicators of active TB transmission in the community.

Globally, TB in children (<15 years old) make up an estimated 10% of all cases, but this is likely underestimated by at least 15–20% due to challenges in diagnostics, surveillance and control [5–10]. Since the predominate focus of TB prevention and control in the Philippines is on the adult population, limited evidence exists regarding prevalence and risk factors for TB in Filipino children. We conducted a cross-sectional paediatric TB prevalence study in the island province of Bohol, Republic of the Philippines [11]. This household-based cluster survey screened 5476 Filipino children for TB and identified a prevalence of positive Mantoux Tuberculin Skin Tests (TST) as high as 29% in some villages. We wanted to know if these aggregated clusters of TST-positive children were associated with distance to health care facilities and resources. We hypothesised that living farther from health care resources contributed to TST positivity, which we used as a proxy for latent TB infection (LTBI).

The primary health care facilities in the Philippines are Rural Health Units (RHUs), which are public community health clinics stationed in each municipality/town. The Department of Health's National Tuberculosis Programme (NTP) through its Regional Offices and Provincial Health Office (PHO), is responsible for TB control at the regional and provincial levels, respectively [12]. The PHO provides support to the RHUs through technical oversight, and the distribution of TB pharmaceutical and diagnostic supplies. RHUs then provide TB diagnostics and treatment via Direct Observed Therapy, Short Course (DOTS) free of cost [12–14]. In Bohol, residents rely on the RHUs for medical care as private health care facilities are expensive and local village clinics, known as barangay health stations (usually manned by a midwife and volunteer barangay health workers), are unable to provide TB specialised care [5]. In 2016, the National Tuberculosis Prevalence Survey found 76% of adult TB cases sought treatment at their RHU, and 72.3% received their anti-TB medications from them [5]. Since RHUs are paramount to the Filipino health infrastructure, they play a critical and salient role in TB identification, prevention and control in the Philippines, especially in areas of poverty.

Based on our concern that geographic isolation is related to increased risk for TB, we conducted a study to determine if paediatric TST-positive prevalence at the village (barangay) level was associated with travel-distance to health care facilities (RHU) or resources for TB control (PHO). To achieve this, we analysed the geospatial relationship between (a) village TST-positive prevalence and the travel-distance to a healthcare facility (PHO or RHU); (b) municipality TST-positive prevalence and the travel-distance to the PHO; and (c) the influence of socioeconomic determinants of health on travel-distance.

## Methods

**Fig. 1.**
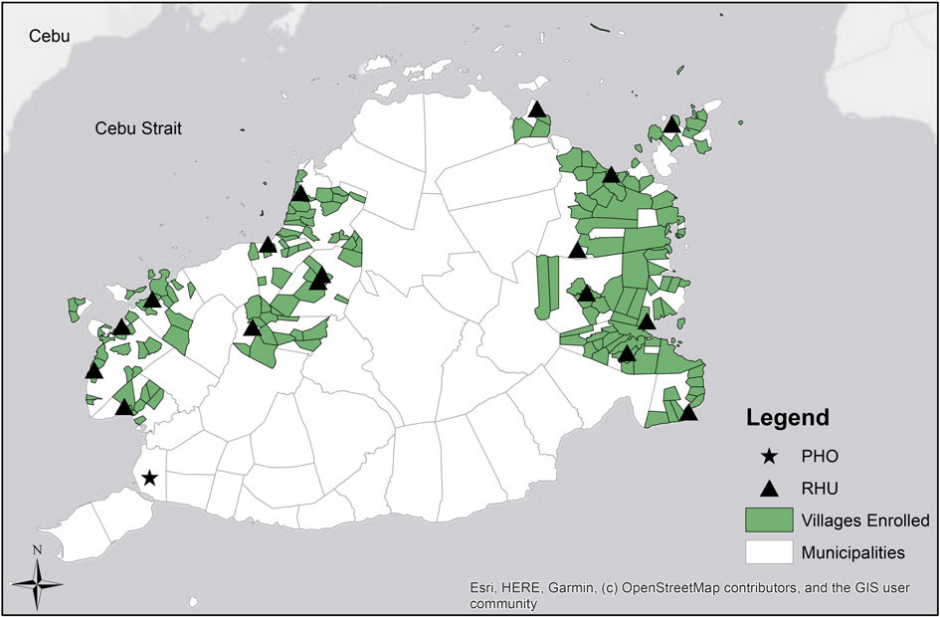
Map of enrolled villages, rural health units and the Provincial Health Office. Map displays the villages (shaded regions), municipal Rural Health Units [RHUs] (triangles) and the Provincial Health Office [PHO] (star) included for analysis in this study. Travel-distance was calculated between the centre of the barangay to RHUs and PHO, respectively. Travel-distance was also calculated between RHUs and the PHO.

This study was approved by the Institutional Review Boards of the University of the Philippines Manila and Baylor College of Medicine (Protocol Number: H-37167). Census data of populations within municipality and village were obtained from the Republic of the Philippines Statistics Authority and the Bohol PHO [15]. For the purposes of this study, we define an ‘island’ as being a piece of land surrounded by a body of water, physically separated but belonging to the geopolitical jurisdiction of a municipality located in mainland Bohol. Data for this study were derived from a large, randomised cluster survey as previously described by Murray *et al*. [11]. The paediatric population was selected using the World Health Organization's EPI methods [16] of population proportion to size cluster sampling among randomly selected households within randomly selected villages between the years 2015 and 2018. Children were screened using TSTs, with an induration of ⩾10 mm indicating LTBI (as recommended by the American Thoracic Society and Centers for Disease Control and Prevention for use in high-risk settings not considered endemic for HIV [17]) and were marked as a positive test result. For this study, we included all TST-positive children in the analysis, which included both LTBI and the 16 active TB disease cases [11]. Locations of the villages, municipal RHUs and the PHO included in this study are indicated in Figure 1. Since the island-wide TST-positive prevalence was found to be 6.5% [11], we used this prevalence as the cut-point and calculated the proportion of villages in each municipality with ⩾6.5% as an indicator of high prevalence of LTBI. We calculated the prevalence of TST-positivity in the municipalities as an indicator of exposure burdens (Table 1) and also calculated TST-positive prevalence by village (Supplemental Table).

**Table 1.** Weighted proportion of villages within municipalities with ⩾6.5% prevalence of TST-positives

Municipality name	Villages with TST-positive prevalence ⩾6.5%/total villages enrolled in study by municipality	Percentage (%)
Pres. Carlos P. Garcia	7/10	70
Bien Unido	4/6	67
Inabanga	14/24	58
Mabini	8/14	57
Maribojoc	4/8	50
Candijay	8/16	50
Ubay	11/23	48
Clarin	5/11	45
Calape	5/11	45
Anda	4/10	40
Sagbayan	2/7	29
Loon	4/24	17
Catigbian	2/13	15
Alicia	0/7	0

**Fig. 2.**
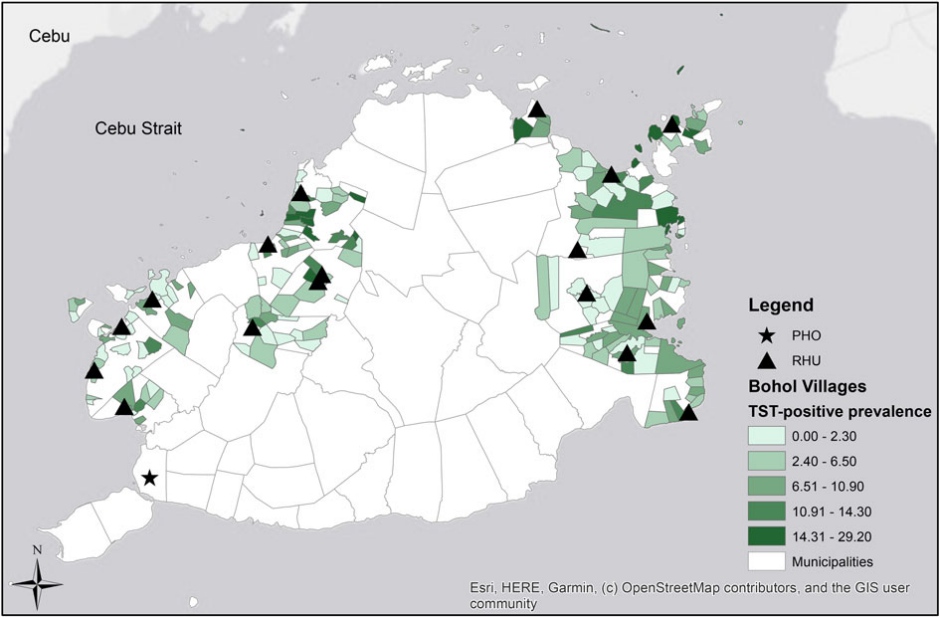
Prevalence of positive TSTs by village.

Political boundaries of municipalities and villages were obtained through PhilGIS^©^ (Philippine GIS Data Clearinghouse, Ozamiz City, Misamis Occidental, Philippines). Point coordinates of the Rural Health Unit locations in each municipality were obtained using a handheld Garmin InReach Explorer^®^ (Garmin Ltd., Olathe, Kansas, USA) and Google Maps^©^ (Google, Mountain View, California, USA) and documented in Microsoft Excel^©^ (Microsoft, Redmond, Washington, USA). Geospatial analysis was conducted using ArcGIS Desktop 10.6^©^ (ESRI, Redlands, California, USA). Choropleth maps were developed to visualize TST-positive prevalence of each participating village to identify which communities had the highest burden of disease (Fig. 2) [18].

**Fig. 3.**
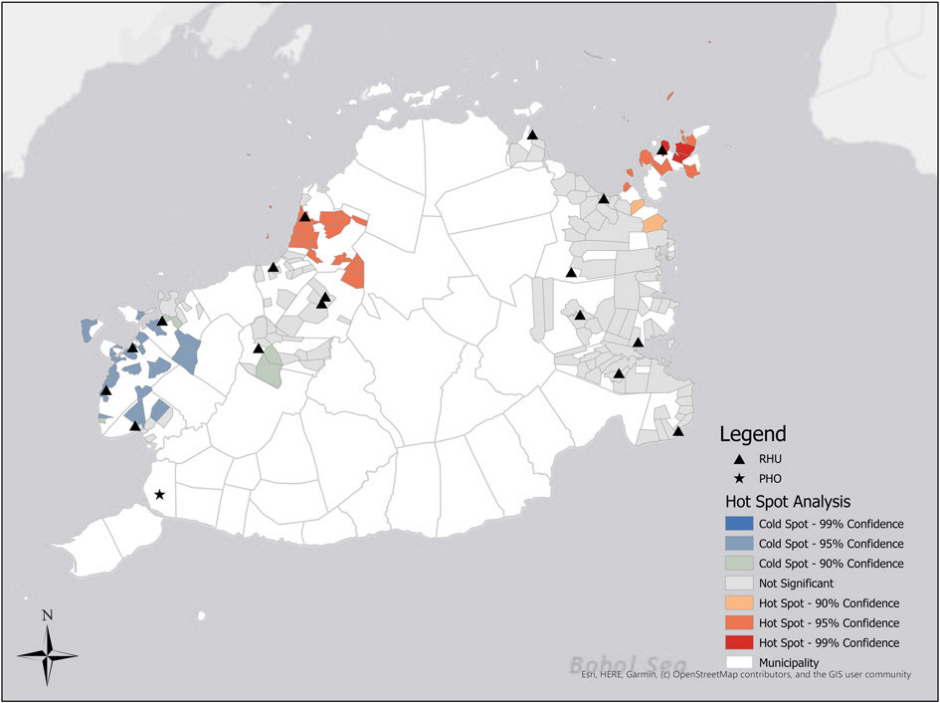
Hot spot analysis of cold and hot spots of TST-positive prevalence aggregated at the village level.

To understand if locations with high prevalence were clusters of TST positivity, we conducted a hot spot analysis of TST-positive prevalence by village using the Optimized Hot Spot Analysis tool in ArcGIS(Fig. 3) [19]. The hot spot analysis uses the Getis-Ord Gi* statistic to calculate each point's *z*-score, *P* value and confidence level to determine if clusters are spatial in nature or due to random chance [20–23]. This tool examines each individual value and then compares to the neighbouring values to determine if there is significant clustering. *z*-Scores that indicate statistically lower prevalence than expected are considered ‘cold spots’ and coloured blue, *z*-scores that statistically high are considered ‘hot spots’ and coloured red, and *z*-scores close to zero are considered ‘not significant’ and coloured grey [22]. Confidence levels are derived from *z*-scores and are based at 90%, 95% and 99% [23].

**Fig. 4.**
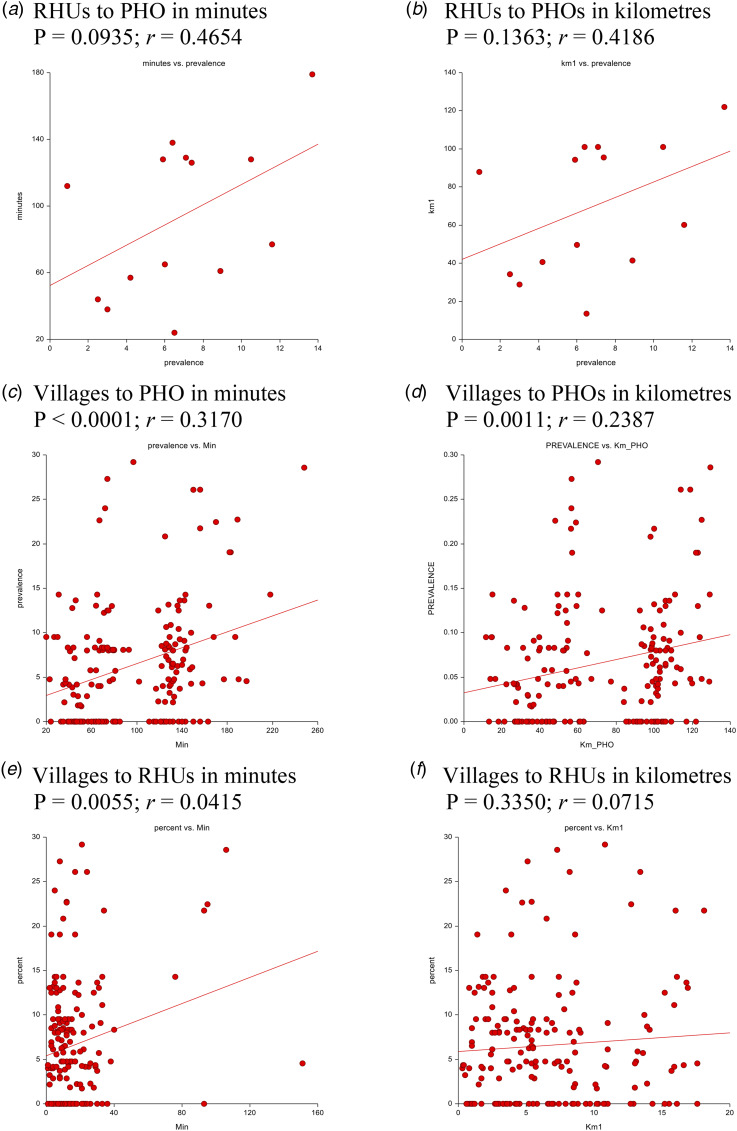
Analysis of association between TST-positive prevalence at the municipality and village level and distance to the PHO and RHU. Association between TST-positive and time-distance in minutes (A) and kilometres (B) was assessed by linear regression between the RHUs and PHO. Association between TST-positive and time-distance in minutes (C) and kilometres (D) was assessed by linear regression between the villages and PHO. Association between TST-positivity and time-distance in minutes (E) and kilometres (F) was assessed by linear regression between the villages and their RHU. *P* values and the correlation coefficients for each analysis is listed below the data description. (A) RHUs to PHO in minutes *P* = 0.0935; *r* = 0.4654, (B) RHUs to PHOs in kilometres *P* = 0.1363; *r* = 0.4186, (C) villages to PHO in minutes *P* < 0.0001; *r* = 0.3170, (D) villages to PHOs in kilometres *P* = 0.0011; *r* = 0.2387, (E) villages to RHUs in minutes *P* = 0.0055; *r* = 0.0415 and (F) villages to RHUs in kilometres *P* = 0.3350; *r* = 0.0715

In a separate analysis, we conducted two regression analyses to analyse predictors of TST positivity in Stata v.14.2^©^ (Statacorp, College Station, TX, USA). First, we used a linear regression analysis to examine the association between distance to health care facilities and TST-positive prevalence by municipality and village (Fig. 4). Distance between points (village to RHU, village to PHO and RHU to PHO) was calculated using travel distance, which we define as the common travel route in physical distance by kilometres (km) and in trip time by minutes (min), known as time-distance, based on the car travel function using Google Maps. To reduce variability in time-distance by car as a result of heavy traffic, car travel times were calculated in one time frame during the middle of the night in Bohol. For the island villages of mainland Bohol who were enrolled in the study (16 total), travel distance to RHUs and the PHO combined Euclidean distance over bodies of water connecting each island's ports and the travel distance from the port to the health care facility on mainland Bohol. All geocoded data including coordinates and distance data were double entered to increase internal reliability and quality assurance.

Lastly, our logistic regression combined socioeconomic determinants of health with our distance data to analyse the role of poverty with access to healthcare providers and TST positivity. Socioeconomic indicators were obtained from the Provincial Government of Bohol's Provincial Planning and Developing Office under their poverty database monitoring system (PDMS) [24]. This survey was created by the Bohol Local Development Foundation in partnership with UNICEF, World Bank and Habitat for Humanity, to be used as a basis for the United Nation's Millennium Development Goals. Poverty indicators were needed for the delivery of targeted interventions to the most disadvantaged communities through housing development, assistance and resources [25, 26]. The PDMS was implemented from 2004–2010 as a province-wide household-based community survey of Bohol to determine the level of poverty in health (water sources, sanitation, child malnutrition and child mortality), economic (income threshold, food threshold and unemployment) and social factors (housing, school dropouts, land tenure and crime incidence) by both municipality and village [25, 27]. Percentages of poverty indicators and TST-positive prevalence were analysed at the village level for comparisons. Each variable was evaluated by univariate analysis to assess their influence in predicting TST positivity. Variables with *P* < 0.25 were entered into the multivariate model, and we used a backwards stepwise approach to eliminate variables with the highest *P* values until the final variables met the *P* < 0.05 cut-off. Interaction terms were evaluated between variables. This approach helped us control for effect modifiers, confounders, identify independent risk factors associated with TST-positivity and estimate variable relationships.

## Results

The distribution of high TST-positive prevalence appeared to be the highest in the municipalities that had the most remote and isolated villages. Among the 14 participating municipalities, President Carlos P. Garcia (7/10, 70%), Bien Unido (4/6, 67%) and Inabanga (14/24, 58%) had the highest proportion of villages with a TST-positive prevalence ⩾6.5% (Table 1). These three municipalities are coastal and contain 13/16 (81%) of the villages located on islands that were randomly selected for inclusion as clusters in the study. The distribution of village TST-positive prevalence (Supplemental Table) was mapped to visualize the varying burden of disease across Bohol (Fig. 2). The choropleth map showed that villages with the highest prevalence were located on the northeast section of Bohol (with the exception of Inabanga on the northwestern side) and were near the coast. Island villages of Gaus (29%), Campamanog (23%) and Bilangbilangan Diot (22%) were in the top ten for highest TST-positive prevalence [11]. The hot spot analysis was consistent with burden of TST positivity. Hot spots clusters were identified in Inabanga, Sagbayan and President Carlos P. Garcia (Fig. 3). Cold spots were located in Calape, Catigbian, Loon and Maribojoc which are in closer proximity to the PHO. Refer to the Supplemental Table for more details.

The linear regression demonstrated time-distance was a stronger variable than Euclidean distance with regard to the effect of travel on TST-positivity (Fig. 4A–F). Analysis of RHUs to the PHO in kilometres (*P* = 0.14, *r* = 0.42) and time-distance (*P* = 0.09, *r* = 0.47) had a weak association with TST-positive prevalence (Fig. 4A and B). However, village distance to the PHO both in kilometres (*P* < 0.001, *r* = 0.24) and time-distance (*P* < 0.0001, *r* = 0.32) had a stronger association and correlation (Fig. 4C and D), with one island village with a prevalence of 29% being located 4.7 h away from the PHO. Lastly, village distance to the municipal RHU in kilometres (*P* = 0.34, *r* = 0.07) did not have a strong effect on TST positivity but distance in time did (*P* = 0.006, *r* = 0.20), indicating a disparity between distance in kilometres and time-distance (Fig. 4E and F).

Since socioeconomic factors can also influence access to transportation and ability to seek healthcare, we wanted to determine these influences in a logistic regression prediction model. A description of socioeconomic health determinants in Bohol is listed in Table 2. The majority of the population is below the income threshold (60.8%) and almost half of the population are below the food threshold (45.8%). Almost 20% of the population do not have access to potable drinking water, experience unemployment and experience total deprivation across all poverty indicators.

**Table 2.** Description of variables included in the univariate logistic regression

Socioeconomic indicators of TST-positive prevalence by villages	Median	Interquartile range
TST-positive prevalence	4.8	9.5
Distance to PHO (km)	91.3	10.1
Distance to PHO (min)	83.5	73.5
Distance to RHU (km)	5.4	5.83
Distance to RHU (min)	11	11.3
*Core Poverty Indicators (by %)*^a^		
Health		
Homes with unsafe source of drinking water	7.9	27.8
Homes with unsanitary toilets	41.6	41.5
Malnourished children	7.1	10.8
Child mortality ages 0–5 years	0	0.4
Economic		
Homes below the income threshold	61.3	33.3
Homes below the food threshold	44.1	34.4
Homes below the meal threshold	0	0.4
Persons who are unemployed	17.9	6.3
Social		
Makeshift housing units	1.9	3.8
Persons who are school dropouts	9.2	9.0
Persons who don't own their household lot	23.7	24.3
Homes affected by crime incidence	0	1.5
*Poverty Database Monitoring System (PDMS) score for total deprivation across all core poverty indicators*	19.5	9.5

a Data derived from Bohol's Poverty Database Monitoring System Survey [26, 27].

Based on univariate analysis, income threshold, school dropouts and total deprivation had an effect on TST-positivity in addition to distance to the PHO in kilometres and minutes. However, due to collinearity of the individual socioeconomic variables, we ran a multivariate logistic regression using total deprivation for all poverty indicators along with geospatial indicators to test for influence on our outcome of villages with TST-positivity ⩾6.5% prevalence (Table 3). Our final model found distance remained significant with travel time in minutes to the PHO with a *P* = 0.03 (OR = 1.01, 95% CI = 1.00–1.02) and our socioeconomic factors were not independently associated with TST positivity. Total deprivation dropped out of the model with a *P* = 0.39. After removing total deprivation from the model, travel time in minutes to the PHO was still significantly associated with villages with a TST positivity ⩾6.5% at *P* = 0.005, OR = 1.01 (95% CI 1.00–1.02). Since, travel time in minutes to the PHO was an independent factor that predicted high TST positivity, we decided to pursue one last analysis to determine what threshold of travel time created a significant increase in TST positive prevalence. Based on univariate analysis, we found children who live an hour or more away from the PHO had three times greater odds of TST positivity compared to children who lives less than an hour away from the PHO (*P* = 0.003, OR = 3.1, 95% CI = 1.5–6.7).

**Table 3.** Results of the univariate and backwards stepwise multivariate logistic regression analyses to determine variables associated with ⩾6.5% TST-positive prevalence

Variables	Univariate analysis		Multivariate model	
Geospatial indicators	Odds ratio (95% CI)	*P* Value	Odds ratio (95% CI)	*P* Value
Distance to PHO (km)	1.01 (1.00–1.02)	0.010^a^	NS	
Distance to PHO (min)	1.01 (1.00–1.02)	0.005^a^	1.01 (1.00–1.02)	0.005
Distance to RHU (km)	0.98 (0.92–1.05)	0.588^a^	NS	
Distance to RHU (min)	1.00 (0.98–1.02)	0.568^a^	NS	
Village was located on an island	2.45 (0.85–7.06)	0.097^a^	NS	
Core poverty indicators				
Health				
Unsafe water source	1.00 (1.00–1.01)	0.325		
Poor toilet sanitation	1.00 (0.99–1.01)	0.731		
Child malnourishment	0.99 (0.96–1.02)	0.476		
Child mortality	0.91 (0.44–1.90)	0.811		
Economic				
Income threshold	1.02 (1.00–1.03)	0.033^b^		
Food threshold	1.01 (0.99–1.03)	0.067^b^		
Meals threshold	1.01 (0.98–1.03)	0.677		
Unemployment	1.02 (0.97–1.06)	0.485		
Social				
Makeshift housing	1.08 (1.00–1.18)	0.056^b^		
School dropouts	1.04 (1.01–1.08)	0.013^b^		
Land tenure status	1.01 (0.99–1.02)	0.311		
Crime incidence	0.97 (0.87–1.08)	0.564		
*Total deprivation*	1.04 (1.00–1.08)	0.044^a^	NS	

NS, not significant.

a Variable included in the multivariate model.

b Not included in the multivariate model due to collinearity with total deprivation.

## Discussion

Our geospatial analysis demonstrates that high paediatric TST-positive prevalence in Bohol, Philippines is associated with living in remote villages. Increased time-distance from villages to the PHO (the government source of resources for TB prevention and control for the province) is a likely barrier as some island villages were more than 4 h away by car and water taxi. Living in remote areas, geographic barriers, poor road conditions, travel by sea, or even concerns about safety, peace and order can make communities hard-to-reach for medical services and continuous care for DOTS. Our analysis highlights that this problem is exacerbated among island villages of Bohol, putting them at higher risk for TST-positivity compared to mainland villages. Island communities have longer and more expensive travel routes to mainland clinics, thereby limiting their medical visits and reducing their access to care. The same is also true for health care providers, where these travel constraints could make it difficult to effectively and regularly deliver healthcare services, supplies and resources to the RHUs and village-level Barangay Health Stations, contributing to service and supply shortages.

TB risk is intertwined with socioeconomic conditions [7]. Risk of paediatric TB infection increases in relationship to age, poverty, housing structure, overcrowding within and outside of the home, geographic location, community prevalence, immune status and malnutrition [3, 6, 28]. Previous research from studies in Bohol found poverty and socioeconomic deprivation to be high in the north and northeast side of the province, which supports our finding of high prevalence of TST-positivity in this area (in President Carlos P. Garcia, Bien Unido and Inabanga) [24]. Canares [29] explained this is due to an ‘urban bias,’ meaning resources and funding, particularly from non-governmental organizations (NGOs), are distributed in areas closest to the capital city of Tagbilaran and are inadequate in municipalities and villages that are farther away. He showed that island and coastal communities are the farthest from the PHO and have minimal to no NGO presence. This funding bias was attributed by Canares [29] to the expenses of transporting healthcare supplies across the island, and in a similar manner, the expense of residents to get to the clinics for the same reasons. MacPherson *et al*. (2019) also showed a geospatial relationship between an increase in TB transmission as there are increases in poorer neighbourhoods that are farther from the TB clinics [30]. We, too, found support for this phenomenon which is known as the ‘inverse case law’ coined by Hart (1971) in the *Lancet* [31]. ‘Inverse case law’ means that people often most in need of specialized care tend to live the farthest from their healthcare providers in rural areas [30, 31]. However, more research is needed to better understand how other socioeconomic factors influence access to TB care and subsequently paediatric transmission.

This study has some noteworthy limitations. First, using Google Maps to calculate time-distance is based on the assumption of travel by private vehicle, limiting our ability to consider multiple modes of travel to a location [20]. As a result, our variable likely underestimates the amount of time it would take to actually get to a clinic by motorbike, water taxi, walking, public transportation (jeepney, bus, tricycle, etc.) or other common means of travel in Bohol. Travel time across water may be subject to inconsistencies based on weather, sea conditions and other variables such as the time it takes for the boat to be filled with passengers before it could leave the port. Second, we were unable to build a predictive model of TST-positivity because PDMS poverty variables were largely collinear, thereby impacting our model building strategies. Due to this collinearity, we opted to use the variable of total deprivation, which incorporates data from all core poverty indicators. Third, our socioeconomic indicators were based on community-level data collected between 2004 and 2010, while our study was conducted around 10 years later from 2015–2018. A more reliable analysis could be produced if we had data available that was temporally collected at the time of the prevalence survey. Fourth, TST positivity was used as a proxy for LTBI, but could overestimate true prevalence in cases of cross-reaction with BCG vaccination. As we discussed in our prior publication [11], cross-reactions should be low since BCG is administered at the time of birth in the Philippines, and TST positivity was significantly associated with older age, indicating true exposure. While interferon gamma release assays (IGRA) might improve specificity, the WHO does not support the use of IGRA in low- and middle-income countries due to insufficient data, prohibitive cost and lacking laboratory infrastructure [32]. It is also possible that prevalence was underestimated since we used the cut-off of ⩾10 mm to determine TST positivity, which would then risk our missing a positive response in malnourished children, where the recommended cut-off is ⩾5 mm [17]. Lastly, we acknowledge distance to health facilities is only one factor associated with TB burden and many other social, economic, political and environmental factors play a role in reduced access to care that are beyond the scope of this study.

In conclusion, we found that distance in time, especially if longer than an hour's drive, was significantly associated with high TST-positive prevalence in children, even when controlling for socioeconomic factors. Improving access to resources for TB prevention and control should be focused on those villages that are more than an hour drive from the PHO. Although many TB control strategies typically focus on older populations, paediatric infections are indicative of adult transmission patterns, and it is critical to identify and treat all age groups exposed. It is paramount for health care providers to begin conducting outreach in these high-risk villages in the form of screening and treatment. We also recommend that local government officials in partnership with public and private health care providers work together to identify ways of overcoming barriers in access to care, such as travel, especially for individuals requiring DOTS.

## Supplementary material

For supplementary material accompanying this paper visit https://doi.org/10.1017/S0950268820000795. click here to view supplementary material
