# Supplementary material for: Geospatial and hot spot analysis of paediatric tuberculosis infection in Bohol, Philippines
Source: Epidemiol Infect. 2020 May 6;148:e89. doi: 10.1017/S0950268820000795 (PMC7286696; doi:10.1017/S0950268820000795)
Supplement: Supplementary file 1 [file S0950268820000795sup001.docx]

**Supplemental Table.** Boholano village names and their corresponding population, paediatric TST-positive prevalence, and their hot spot analysis result derived using the Getis-Ord Gi* statistic.

| Municipality Name | Village Name | Village Population | TST-positive prevalence | Cold or Hot Spot with CI |
| --- | --- | --- | --- | --- |
| Alicia | Cayacay | 1,713 | 0.0% | Not Significant |
| Alicia | Katipunan | 2,230 | 3.7% | Not Significant |
| Alicia | La Hacienda | 3,710 | 2.2% | Not Significant |
| Alicia | Napo | 1,255 | 0.0% | Not Significant |
| Alicia | Poblacion | 4,064 | 0.0% | Not Significant |
| Alicia | Progreso | 1,019 | 0.0% | Not Significant |
| Alicia | Putlongcam | 1,578 | 0.0% | Not Significant |
| Anda | Almaria | 392 | 0.0% | Not Significant |
| Anda | Bacong | 2,289 | 13.2% | Not Significant |
| Anda | Badiang | 1,277 | 9.1% | Not Significant |
| Anda | Candabong | 2,297 | 6.3% | Not Significant |
| Anda | Linawan | 987 | 6.4% | Not Significant |
| Anda | Poblacion | 1,295 | 3.2% | Not Significant |
| Anda | Santa Cruz | 1,123 | 5.6% | Not Significant |
| Anda | Suba | 1,125 | 2.9% | Not Significant |
| Anda | Talisay | 1,048 | 8.3% | Not Significant |
| Anda | Virgen | 1,428 | 8.1% | Not Significant |
| Bien Unido | **Bilangbilangan Dako** | **1,920** | **0.0%** | **Not Significant** |
| Bien Unido | **Bilangbilangan Diot** | **845** | **21.7%** | **Not Significant** |
| Bien Unido | Mandawa | 2,328 | 8.5% | Not Significant |
| Bien Unido | Nueva Esperanza | 2,205 | 20.8% | Not Significant |
| Bien Unido | Poblacion | 3,082 | 4.0% | Not Significant |
| Bien Unido | Tuboran | 955 | 9.5% | Not Significant |
| Calape | Abucayan Sur | 786 | 8.3% | Not Significant |
| Calape | Bentig | 1,797 | 0.0% | Not Significant |
| Calape | Bonbon | 1,222 | 0.0% | Cold Spot with 90% Confidence |
| Calape | Cabayugan | 880 | 4.2% | Cold Spot with 95% Confidence |
| Calape | Labuon | 562 | 8.0% | Cold Spot with 95% Confidence |
| Calape | Lawis | 617 | 0.0% | Cold Spot with 95% Confidence |
| Calape | Liboron | 1,434 | 9.5% | Cold Spot with 95% Confidence |
| Calape | Mandaug | 1,451 | 8.3% | Not Significant |
| Calape | San Isidro | 2,412 | 0.0% | Not Significant |
| Calape | Santa Cruz | 2,401 | 0.0% | Cold Spot with 90% Confidence |
| Calape | Sojoton | 664 | 9.1% | Cold Spot with 95% Confidence |
| Candijay | Abihilan | 1,327 | 0.0% | Not Significant |
| Candijay | Boyo-An | 1,632 | 0.0% | Not Significant |
| Candijay | Cambane | 665 | 4.5% | Not Significant |
| Candijay | Can-Olin | 1,637 | 0.0% | Not Significant |
| Candijay | Canawa | 2,466 | 0.0% | Not Significant |
| Candijay | Cogtong | 3,220 | 6.5% | Not Significant |
| Candijay | La Union | 1,691 | 0.0% | Not Significant |
| Candijay | Luan | 937 | 12.5% | Not Significant |
| Candijay | Lungsoda-An | 1,853 | 10.4% | Not Significant |
| Candijay | Panadtaran | 1,511 | 8.3% | Not Significant |
| Candijay | Panas | 1,705 | 8.0% | Not Significant |
| Candijay | Poblacion | 4,320 | 8.5% | Not Significant |
| Candijay | San Isidro | 1,380 | 4.0% | Not Significant |
| Candijay | Tambongan | 1,830 | 3.7% | Not Significant |
| Candijay | Tubod | 2,052 | 8.0% | Not Significant |
| Candijay | Tugas | 1,214 | 12.5% | Not Significant |
| Catigbian | Alegria | 1,408 | 7.1% | Not Significant |
| Catigbian | Ambuan | 1,259 | 3.8% | Cold Spot with 90% Confidence |
| Catigbian | Bagtic | 1,123 | 0.0% | Not Significant |
| Catigbian | Bongbong | 757 | 0.0% | Cold Spot with 90% Confidence |
| Catigbian | Cambailan | 933 | 1.7% | Not Significant |
| Catigbian | Candumayao | 1,680 | 2.9% | Not Significant |
| Catigbian | Causwagan Norte | 1,715 | 3.0% | Not Significant |
| Catigbian | Haguilanan | 1,184 | 2.2% | Not Significant |
| Catigbian | Kang-Iras | 831 | 4.2% | Not Significant |
| Catigbian | Mahayag Norte | 722 | 1.8% | Not Significant |
| Catigbian | Mantasida | 1,025 | 0.0% | Not Significant |
| Catigbian | Poblacion | 1,752 | 12.8% | Not Significant |
| Catigbian | Triple Union | 1,223 | 1.9% | Not Significant |
| Clarin | Bacani | 1,208 | 4.0% | Not Significant |
| Clarin | Bogtongbod | 1,377 | 0.0% | Not Significant |
| Clarin | Buacao | 797 | 0.0% | Not Significant |
| Clarin | Caboy | 571 | 8.3% | Not Significant |
| Clarin | Candajec | 932 | 14.3% | Not Significant |
| Clarin | Danahao | 856 | 0.0% | Not Significant |
| Clarin | Mataub | 700 | 8.0% | Not Significant |
| Clarin | Nahawan | 2,208 | 8.0% | Not Significant |
| Clarin | Poblacion Centro | 1,234 | 13.0% | Not Significant |
| Clarin | Poblacion Sur | 1,159 | 4.0% | Not Significant |
| Clarin | Villaflor | 345 | 4.5% | Not Significant |
| Inabanga | Anonang | 721 | 29.2% | Hot Spot with 95% Confidence |
| Inabanga | Badiang | 1,083 | 24.0% | Hot Spot with 95% Confidence |
| Inabanga | Baogo | 1,252 | 4.5% | Hot Spot with 95% Confidence |
| Inabanga | Cagawasan | 1,290 | 13.0% | Hot Spot with 95% Confidence |
| Inabanga | Cambitoon | 919 | 12.5% | Hot Spot with 95% Confidence |
| Inabanga | Cogon | 865 | 12.5% | Hot Spot with 95% Confidence |
| Inabanga | **Cuaming** | **2,826** | **22.4%** | **Hot Spot with 90% Confidence** |
| Inabanga | Dagohoy | 1,310 | 27.3% | Hot Spot with 95% Confidence |
| Inabanga | Ilaud | 954 | 4.3% | Hot Spot with 95% Confidence |
| Inabanga | Liloan Norte | 1,490 | 4.8% | Hot Spot with 95% Confidence |
| Inabanga | Liloan Sur | 954 | 7.7% | Hot Spot with 95% Confidence |
| Inabanga | Lomboy | 589 | 11.1% | Hot Spot with 95% Confidence |
| Inabanga | Lutao | 1,173 | 4.8% | Hot Spot with 95% Confidence |
| Inabanga | Mabuhay | 383 | 19.0% | Hot Spot with 95% Confidence |
| Inabanga | Nabuad | 1,804 | 0.0% | Hot Spot with 95% Confidence |
| Inabanga | Ondol | 1,122 | 0.0% | Not Significant |
| Inabanga | Poblacion | 930 | 0.0% | Hot Spot with 95% Confidence |
| Inabanga | Riverside | 260 | 14.3% | Hot Spot with 95% Confidence |
| Inabanga | San Jose | 1,566 | 0.0% | Hot Spot with 95% Confidence |
| Inabanga | Santo Rosario | 997 | 9.1% | Hot Spot with 95% Confidence |
| Inabanga | Sua | 554 | 21.7% | Hot Spot with 95% Confidence |
| Inabanga | Tambook | 490 | 14.3% | Hot Spot with 95% Confidence |
| Inabanga | Tungod | 1,089 | 0.0% | Hot Spot with 95% Confidence |
| Inabanga | Ubujan | 1,064 | 0.0% | Not Significant |
| Loon | Bahi | 367 | 0.0% | Cold Spot with 95% Confidence |
| Loon | Basac | 1,414 | 0.0% | Cold Spot with 95% Confidence |
| Loon | Bugho | 285 | 0.0% | Cold Spot with 95% Confidence |
| Loon | **Cabacongan** | **1,080** | **0.0%** | **Cold Spot with 95% Confidence** |
| Loon | Calayugan Norte | 737 | 0.0% | Cold Spot with 95% Confidence |
| Loon | Canhangdon Occidental | 848 | 8.3% | Cold Spot with 95% Confidence |
| Loon | Canmaag | 404 | 0.0% | Cold Spot with 95% Confidence |
| Loon | Cansuagwit | 291 | 0.0% | Cold Spot with 95% Confidence |
| Loon | Cantam-Is Baslay | 495 | 0.0% | Cold Spot with 95% Confidence |
| Loon | Catagbacan Handig | 994 | 0.0% | Cold Spot with 95% Confidence |
| Loon | Catagbacan Sur | 973 | 0.0% | Cold Spot with 95% Confidence |
| Loon | Cogon Norte | 1,907 | 4.2% | Cold Spot with 95% Confidence |
| Loon | Cuasi | 1,115 | 0.0% | Cold Spot with 90% Confidence |
| Loon | Genomoan | 362 | 13.6% | Cold Spot with 95% Confidence |
| Loon | Lintuan | 913 | 4.2% | Cold Spot with 95% Confidence |
| Loon | **Looc** | **1,070** | **8.3%** | **Cold Spot with 95% Confidence** |
| Loon | Moto Norte | 1,369 | 0.0% | Cold Spot with 95% Confidence |
| Loon | Moto Sur | 1,225 | 4.3% | Cold Spot with 95% Confidence |
| Loon | Napo | 1,342 | 0.0% | Cold Spot with 95% Confidence |
| Loon | **Pantudlan** | **808** | **4.8%** | **Cold Spot with 95% Confidence** |
| Loon | Pondol | 1,476 | 0.0% | Cold Spot with 95% Confidence |
| Loon | Tangnan | 867 | 4.2% | Cold Spot with 95% Confidence |
| Loon | Tubuan | 285 | 0.0% | Cold Spot with 95% Confidence |
| Loon | Ubojan | 486 | 8.3% | Cold Spot with 95% Confidence |
| Mabini | Abaca | 2,782 | 4.3% | Not Significant |
| Mabini | Baybayon | 1,886 | 6.1% | Not Significant |
| Mabini | Bulawan | 789 | 7.3% | Not Significant |
| Mabini | Cabidian | 1,348 | 10.6% | Not Significant |
| Mabini | Cawayanan | 2,035 | 6.1% | Not Significant |
| Mabini | Lungsoda-An | 1,309 | 2.2% | Not Significant |
| Mabini | Minol | 1,721 | 9.3% | Not Significant |
| Mabini | Paraiso | 773 | 10.0% | Not Significant |
| Mabini | Poblacion II | 2,068 | 6.9% | Not Significant |
| Mabini | San Isidro | 1,803 | 7.0% | Not Significant |
| Mabini | San Jose | 1,848 | 5.9% | Not Significant |
| Mabini | San Roque | 2,981 | 6.3% | Not Significant |
| Mabini | Tangkigan | 1,788 | 8.8% | Not Significant |
| Mabini | Valaga | 1,010 | 8.0% | Not Significant |
| Maribojoc | Bayacabac | 1,601 | 14.3% | Not Significant |
| Maribojoc | Dipatlong | 1,562 | 9.5% | Not Significant |
| Maribojoc | Jandig | 897 | 9.5% | Cold Spot with 95% Confidence |
| Maribojoc | Poblacion | 2,298 | 0.0% | Not Significant |
| Maribojoc | San Isidro | 525 | 4.8% | Cold Spot with 95% Confidence |
| Maribojoc | San Roque | 1,177 | 4.8% | Not Significant |
| Maribojoc | San Vicente | 1,115 | 9.5% | Not Significant |
| Maribojoc | Tinibgan | 614 | 0.0% | Cold Spot with 95% Confidence |
| Pres. Carlos P. Garcia | **Aguining** | **2,294** | **9.5%** | **Hot Spot with 95% Confidence** |
| Pres. Carlos P. Garcia | **Bonbonon** | **1,286** | **9.5%** | **Hot Spot with 99% Confidence** |
| Pres. Carlos P. Garcia | **Butan** | **626** | **14.3%** | **Hot Spot with 99% Confidence** |
| Pres. Carlos P. Garcia | **Campamanog** | **1,560** | **22.7%** | **Hot Spot with 95% Confidence** |
| Pres. Carlos P. Garcia | **Gaus** | **1,365** | **28.6%** | **Hot Spot with 95% Confidence** |
| Pres. Carlos P. Garcia | **Poblacion** | **2,700** | **19.0%** | **Hot Spot with 99% Confidence** |
| Pres. Carlos P. Garcia | **Saguise** | **745** | **4.8%** | **Hot Spot with 95% Confidence** |
| Pres. Carlos P. Garcia | **Tilmobo** | **197** | **4.5%** | **Not Significant** |
| Pres. Carlos P. Garcia | **Tugnao** | **1,309** | **19.0%** | **Hot Spot with 99% Confidence** |
| Pres. Carlos P. Garcia | **Villa Milagrosa** | **1,273** | **4.8%** | **Hot Spot with 95% Confidence** |
| Sagbayan | Canmaya Centro | 1,317 | 12.2% | Not Significant |
| Sagbayan | Langtad | 570 | 5.7% | Hot Spot with 95% Confidence |
| Sagbayan | Libertad Norte | 316 | 0.0% | Not Significant |
| Sagbayan | Poblacion | 3,945 | 5.8% | Not Significant |
| Sagbayan | Sagbayan Sur | 1,011 | 5.8% | Not Significant |
| Sagbayan | San Antonio | 852 | 2.9% | Not Significant |
| Sagbayan | Santa Cruz | 985 | 22.6% | Not Significant |
| Ubay | Achila | 1,276 | 0.0% | Not Significant |
| Ubay | Bay-Ang | 1,656 | 4.2% | Not Significant |
| Ubay | Benliw | 2,223 | 0.0% | Hot Spot with 90% Confidence |
| Ubay | Biabas | 2,573 | 4.3% | Not Significant |
| Ubay | Bood | 2,717 | 0.0% | Not Significant |
| Ubay | Cagting | 1,597 | 26.1% | Not Significant |
| Ubay | Calanggaman | 1,623 | 0.0% | Not Significant |
| Ubay | Camambugan | 2,251 | 0.0% | Not Significant |
| Ubay | Casate | 2,512 | 8.0% | Not Significant |
| Ubay | Cuya | 516 | 13.0% | Not Significant |
| Ubay | Fatima | 3,235 | 13.0% | Not Significant |
| Ubay | Guintabo-An | 686 | 0.0% | Not Significant |
| Ubay | Humayhumay | 1,708 | 4.8% | Not Significant |
| Ubay | Imelda | 1,761 | 13.6% | Not Significant |
| Ubay | Katarungan | 1,524 | 13.6% | Not Significant |
| Ubay | Lomangog | 2,025 | 4.8% | Not Significant |
| Ubay | Pag-Asa | 1,168 | 8.7% | Not Significant |
| Ubay | Poblacion | 3,633 | 6.5% | Not Significant |
| Ubay | San Pascual | 3,127 | 2.3% | Not Significant |
| Ubay | Sentinila | 969 | 26.1% | Hot Spot with 95% Confidence |
| Ubay | Tapon | 2,481 | 10.9% | Not Significant |
| Ubay | Tipolo | 2,456 | 0.0% | Not Significant |
| Ubay | Union | 2,332 | 14.3% | Hot Spot with 90% Confidence |

* Names in bold font represent the island municipality and villages surrounding mainland Bohol
